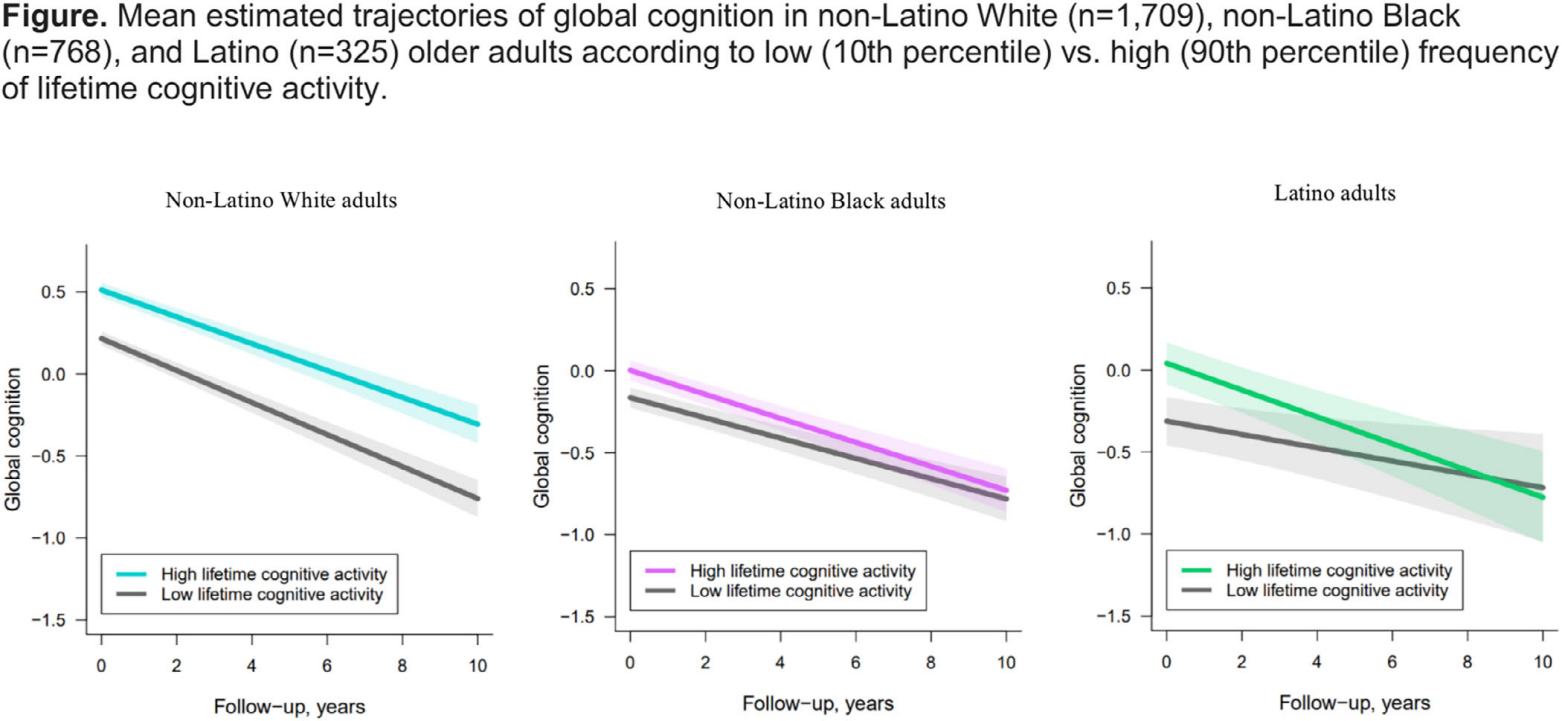# Association of lifetime cognitive activities and resources with change in cognition among US‐based White, Black, and Latino older adults

**DOI:** 10.1002/alz70860_105092

**Published:** 2025-12-23

**Authors:** Mayra L. Estrella, Maude Wagner, Robert S. Wilson, Lisa L. Barnes, David A. A. Bennett, David X. Marquez, Melissa Lamar

**Affiliations:** ^1^ Rush Alzheimer's Disease Center, Rush University Medical Center, Chicago, IL, USA; ^2^ Department of Internal Medicine, Rush University Medical Center, Chicago, IL, USA; ^3^ Rush Alzheimer's Disease Center, Rush University Medical Center, Chicago, IL, USA; ^4^ Department of Neurological Sciences, Rush University Medical Center, Chicago, IL, USA; ^5^ Department of Psychiatry and Behavioral Sciences, Rush University Medical Center, Chicago, IL, USA; ^6^ Department of Kinesiology and Nutrition, University of Illinois Chicago, Chicago, IL, USA

## Abstract

**Background:**

Participation in cognitively stimulating activities and access to cognitive resources have been linked to reduced late‐life cognitive decline in older non‐Hispanic white adults. However, research across racially and ethnically diverse populations remains limited.

**Method:**

We included 1,709 non‐Hispanic White, 768 non‐Hispanic Black, and 325 Latino older adults (≈77yrs at baseline; 76% women) from Rush Alzheimer's Disease Center cohort studies. Participants were dementia‐free at baseline and completed at least two cognitive evaluations (average follow‐up ≈8±5 years). At baseline, participants reported the frequency of current (late‐life) and past (childhood, young adulthood, middle age) cognitive activity, from which 4 composite measures and a total lifetime score were derived. Participants also reported past (childhood, middle‐age) cognitive resources in the home from which 2 composite measures and a total score were derived. For each of the three diverse subgroups, linear mixed‐effects models examined the associations of cognitive activity and cognitive resources (lifetime/total and life‐stage specific), separately, with global cognitive change, adjusting for age, sex, and education (plus interview language for Latino participants), and their interactions with time.

**Result:**

For each diverse subgroup, more frequent cognitive activity and greater access to cognitively stimulating resources, across all life‐stages, were associated with higher initial global cognitive level (all *p* ≤.0001; Figure). However, only lifetime cognitive activity was associated with cognitive decline. Specifically, in White adults, more frequent lifetime cognitive activity was associated with slower cognitive decline (estimate=0.012, standard error [SE]=0.006, *p* = 0.04). Contrastingly, in Latino adults, higher lifetime cognitive activity was associated with faster cognitive decline (estimate=‐0.024, SE=0.010, *p* = 0.01; Figure), primarily driven by cognitive activity in childhood (estimate=‐0.016, SE=0.007, *p* = 0.02) and young adulthood (estimate=‐0.020, SE=0.007, *p* = 0.01). These results persisted after adjusting for depressive symptoms, chronic conditions, and nativity. Cognitive activity was not associated with cognitive change in Black adults. Cognitive resources were not associated with cognitive change in any subgroup.

**Conclusion:**

Findings highlight racial and ethnic differences in the relationships of cognitive activity with cognitive change, emphasizing the utility of traditional measures of cognitive activities for some, but not all, older adults (e.g., older Black adults). Future studies may aim to develop novel cognitive activity or cognitive resource measures.